# The optimal co-insurance rate for outpatient drug expenses of Iranian health insured based on the data mining method

**DOI:** 10.1186/s12939-023-02065-4

**Published:** 2024-02-08

**Authors:** Shekoofeh Sadat Momahhed, Sara Emamgholipour Sefiddashti, Behrouz Minaei, Maryam Arab

**Affiliations:** 1 Department of Health Management and Economics, School of Public Health, Tehran University of Medical Sciences, Tehran, Iran; 2 School of Computer Engineering, Iran University of Science and Technology, Tehran, Iran; 3 National Center for Health Insurance Research (Iran Health Insurance Organization), Tehran, Iran

**Keywords:** Health insurance, Data mining, Optimal co-insurance

## Abstract

**Objective:**

A more equal allocation of healthcare funds for patients who must pay high costs of care ensures the welfare of society. This study aimed to estimate the optimal co-insurance for outpatient drug costs for health insurance.

**Setting:**

The research population includes outpatient prescription claims made by the Health Insurance Organization that outpatient prescriptions in a timely manner in 2016, 2017, 2018, and 2019 were utilized to calculate the optimal co-insurance. The study population was representative of the research sample.

**Design:**

At the secondary level of care, 11 features of outpatient claims were studied cross-sectionally and retrospectively using data mining. Optimal co-insurance was estimated using Westerhut and Folmer's utility model.

**Participants:**

One hundred ninety-three thousand five hundred fifty-two individuals were created from 21 776 350 outpatient claims of health insurance. Because of cost-sharing, insured individuals in a low-income subsidy plan and those with refractory diseases were excluded.

**Results:**

Insureds were divided into three classes of low, middle, and high risk based on IQR and were separated to three clusters using the silhouette coefficient. For the first, second, and third clusters of the low-risk class, the optimal co-insurance estimates are 0.81, 0.76, and 0.84, respectively. It was equal to one for all middle-class clusters and 0.38, 0.45, and 0.42, respectively, for the high-risk class. The insurer's expenses were altered by $3,130,463, $3,451,194, and $ 1,069,859 profit for the first, second, and third clusters, respectively, when the optimal co-insurance strategy is used for the low-risk class. For middle risks, it was US$29,239,815, US$13,863,810, and US$ 14,573,432 while for high risks, US$4,722,099, US$ 6,339,317, and US$19,627,062, respectively.

**Conclusions:**

These findings can improve vulnerable populations' access to costly medications, reduce resource waste, and help insurers distribute funds more efficiently.

## Strengths and limitations of this study

- ➢ The estimation of optimal co-insurance can be used across the entire nation because it is based on data from the national health insurance system, and the research sample is matched to the population.
- ➢ Researchers and policymakers may be able to compare the findings of this study with those of other studies because the results of estimating co-insurance for each cluster in each class are expressed as a percentage (rate).
- ➢ The health insurance company ensures that they do not pay for all medications in their outpatient claims.
- ➢ The results are unreliable because the income information for each insured was based on an artificial neural network's assessment, and was therefore approximate.

## Introduction

In the 20^th^ century, governments worldwide pursued the goal of achieving universal health coverage. However, this endeavor was accompanied by formidable challenges, notably resource constraints and escalating healthcare costs. Simultaneously, health systems strove to shield individuals from the financial burden associated with illness [1]. In 2000, the World Health Organization underscored the paramount importance of prepayment proportions in healthcare financing, emphasizing the reduction of out-of-pocket expenses and the alignment of prepayments with individuals' financial capabilities to prevent impoverishment [1]. Equitable healthcare financing entails that those with greater means contribute proportionally [2] and that healthcare expenditures, particularly in the domain of pharmaceuticals, do not impede essential life needs [3]. Ensuring affordable and accessible medicines plays a critical role in healthcare delivery, significantly influencing both therapeutic and preventive services [4]. Facilitating access to essential medications serves as a fundamental performance indicator for healthcare systems, mitigating the burden of illness and extending life expectancy [5].

Recently, the absolute and relative costs of medications have emerged as a central concern for policymakers globally [6]. According to the 2011 World Health Organization report on drug status, medication costs constitute a range of 1.41% to 1.63% of the gross domestic product, with variations depending on regional disparities and income strata, manifesting a significant difference of 0.2%–3.8% of GDP between these two bounds. Notably, medication costs typically represent the second or third largest component of overall healthcare expenses in all countries following hospital admissions and physician visits [7, 8]. Moreover, in many economically disadvantaged nations, pharmaceutical expenses account for a substantial share of total healthcare expenditure, ranging between 20% and 40%, in contrast to 10% to 20% in more economically advanced countries [9]. Generally, low-income countries allocate a greater proportion of their total healthcare budget to medications, averaging 24.9% of global healthcare expenditure but varying from 7.7% to 67.7% across nations [8].

Medications have consistently maintained distinct significance as essential commodities and fundamental necessities [10]. From an economic perspective, particularly within the domain of health economics, the level of pharmaceutical utilization is a pivotal indicator within the healthcare systems of nations, significantly contributing to the overall rise in healthcare expenditures and imposing a substantial societal and individual burden [11]. In this context, resource constraints often impede the ability to procure an adequate supply of medications to meet the needs of disadvantaged populations. Impediments to access, including prescription expenses, household income, and other pertinent considerations, further compound this predicament [12]. The extent of patient contributions to prescription expenses varies across drug categories in the United States, with the government offering separate subsidies to support specific population segments. In this nation, medication expenses constitute 10% of healthcare costs and account for 31% of out-of-pocket expenses. Medication utilization in the United States is subject to various determinants, including the aging population, insurance coverage, and, to a certain extent, individual financial capacity [13]. Across different countries, patients bear varying out-of-pocket drug expenses, with the highest percentages observed in Finland (36%), Canada (28%), South Korea (27%), Sweden (22%), Germany (15%), Spain (6%), and Iran (30%) [14].

The payment of medication costs, coupled with the associated technical fee for each prescription, is an obligatory requirement for pharmacies to dispense drugs to patients. Consequently, a patient's ability to pay is a pivotal factor that influences access to prescribed medications. Notably, a survey revealed that over the course of a year, 13% of individuals with Medicaid or public insurance and 5% of those with private insurance refrained from obtaining prescriptions because of financial constraints. This is despite the insurance mechanisms being designed to mitigate patients' out-of-pocket expenses. Furthermore, the report highlights that 22% of individuals with insurance coverage and 45% of those lacking insurance altogether avoid visiting pharmacies because of the unaffordability of prescription drugs [15].

Elevated service costs, particularly pertaining to prescription expenses, possess the potential to dissuade individuals from seeking healthcare, thereby exposing them to financial hardship [16]. Moreover, the cost of medications is increasing at a rate surpassing the per capita medication budgets [13]. When healthcare insurance aims to facilitate access to care, copayments must be meticulously designed [17]. The seminal RAND experiment of the 1970s demonstrated that co-insurance models led to reduced healthcare spending, with an estimated price elasticity of -0.2, signifying that a 1% price increase results in a 0.2% decline in healthcare demand [18].

Numerous determinants, including age, sex, and socioeconomic status, play influential roles in shaping individuals' patterns of healthcare utilization. The affordability of health care is significantly contingent on the extent of insurance coverage. Additionally, health insurance organizations are subject to diverse factors, including patient preferences, service quality, financial considerations, socioeconomic circumstances, urban or rural settings, and the nature of medical conditions [19].

Empirical evidence illuminates the imperative role of cost-sharing mechanisms for both patients and insurers within the healthcare realm. Governmental responsibility is underscored in light of the burgeoning costs associated with medications and the pressing need for accessible high-quality drugs. Co-insurance has emerged as a viable strategy for cost management and the support of vulnerable demographic segments, aligned with empirical findings [17]. Co-insurance mandates that patients contribute a proportion of their treatment expenses, with two prevailing primary models: fixed and variable. Fixed co-insurance constitutes 30% of outpatient expenses in Iran, with health insurance organizations covering the remaining 70%, irrespective of service type or demographic attributes. By contrast, variable co-insurance introduces a dynamic element, adjusting the patient's share based on the cost of care, thereby promoting cost-effectiveness and equitable access [20].

It is imperative for all stakeholders, including insured individuals, patients under health insurance, and health insurance organizations, to ascertain the optimal level of co-insurance for various categories of pharmaceuticals, encompassing both acute and chronic treatments, while considering behavioral responses. Co-insurance, whether fixed or variable, can lead to excessive costs or resource inefficiency, if not underpinned by equitable support, comprehensive cost coverage, and risk-sharing principles contingent upon individuals' behavioral choices [21].

As a fundamental imperative, governments must allocate healthcare resources in a manner that enhances the fairness of the financial participation index, thereby expanding access to healthcare services and curbing the percentage of low-income and vulnerable households that grapple with exorbitant health-related expenses. The findings underscore that the proportion of households incurring healthcare expenses and the number of households at risk of vulnerability due to unaffordable healthcare costs should not escalate [22].

This study provides valuable insights into the capacity of health insurance organizations to adapt their co-insurance policies in response to dynamic factors, such as fluctuating drug prices, evolving healthcare costs, and the specific attributes of insured individuals. These organizations often grapple with resource limitations, necessitating strategic resource allocation to mitigate the financial burden incurred by individuals subjected to high co-insurance rates [22]. This research addresses situations where individuals may not be fully attuned to the economic implications of their healthcare decisions, potentially resulting in excessive resource utilization. The primary focus of this study is the optimization of co-insurance structures to concurrently achieve two central objectives: cost containment and preservation of unimpeded access to healthcare services without imposing significant financial constraints [21].

This study contributes significantly to the existing literature by introducing a novel and innovative perspective. It fills a critical void as prior studies predominantly concentrated on the adequacy of co-insurance rates within the context of drugs designed for specific medical conditions. Consequently, this study represents an original and distinct contribution by incorporating a comprehensive analysis of optimal co-insurance rates [21].

This study adopted a multifaceted approach, entailing a meticulous examination of outpatient prescription patterns among insured patients. This analysis encompasses an array of factors, including risk profiles, demographic characteristics, and financial considerations, across diverse categories of medications, spanning both acute and chronic treatments. The overarching objective is to delineate distinct clusters of insured individuals, based on their distinctive characteristics and risk profiles. Subsequently, the research endeavors to identify the most fitting variable co-insurance structures for each cluster, carefully tailored to their respective risk profiles, demographics, and financial implications of their outpatient medication requirements, spanning both acute and chronic health conditions. This holistic evaluation extends to the supply side, where the study quantifies the potential resource savings and cost dynamics realized through the implementation of these optimized variable co-insurance models within the health insurance system over a predefined temporal framework.

## Methods

### Data, participants, and eligibility criteria

There were no missing data points in the collection of data from the health insurance company, indicating that the organization itself carried out extensive data cleaning and validation processes on the dataset. Ensuring the correctness, completeness, and dependability of the dataset, this rigorous data-cleaning method complies with industry requirements. Thus, the data is quite trustworthy and appropriate for thorough analysis and study in the field of healthcare. Also, according to the data gathered, every four years (2016–2019), 21 776 350 outpatient prescription claims were isolated from one another based on the particular codes and eleven characteristics of the outpatient prescription. The researcher converted the outpatient prescriptions to each insured using these identification codes. With Python software, basic mathematical operations such as addition and multiplication were performed by applying the necessary codes.

The National Health Insurance Organization outpatient prescription claims from 2016 to 2019 were included in the study population. These claims data were used because the demographic data and prescriptions from 2016 were accurately registered. In this study, the population is represented by the research sample, and the data of the sample—each outpatient prescription claim in full throughout the course of the years 2016, 2017, 2018, and 2019—were used to estimate the optimal co-insurance. Additionally, data from the Statistical Center of Iran were used to acquire income information from the insured. Iranians with health insurance who receive insurance subsidies for low-income individuals as well as those insured with insurance coverage for refractory disease from the target sample due to their zero cost sharing in insurance programs were eliminated from the sample in accordance with the topic of cost sharing and the main objective of the research.

### Study design and setting

Health insurance outpatient claims were polled to gather the necessary statistical information for the current study, which is cross-sectional, retrospective, based on secondary care and secondary health insurance data. By consulting with the National Health Insurance Research Center and looking through health insurance outpatient claims, the necessary data were gathered. Excel 2016 software was used to gather data from the currently accessible sources. The Information Department of the Health Insurance Research Center thereafter offered a list of medications covered by health insurance. The World Health Organization defines chronic disease as having a persistent duration, typically advancing slowly, and not being passed from person to person; acute disease is a term used to describe a disorder that often manifests fast and goes away in under six months and was used by three specialists to divide medicines into two drug groups for acute and chronic diseases [23]. In light of the problem of overlapping prescriptions, experts believe that when drugs are suggested for both acute and chronic conditions, they are included in a group that is more commonly used and prescribed for that disease (acute or chronic) [14].

### Variables

Among the features that can be mentioned for all insured in each cluster throughout a four-year period are demographics: gender, age (categorized as [1-10], [10-20], [20-30], [30-40], [40-50], [50–60], and ≥ 60), main (being a householder or family member), total average number of medicines, total average number of medicines for acute and chronic disease, total average insurance paid, total average franchise (co-insurance cost paid by insured), total average number of prescriptions, total average insurance paid and deductions, total average income (estimated income for each insured using artificial neural network), and total average deduction (deductions per prescription).

### Statistical analysis

By implementing advancements made with growing attention, the majority of insurance firms have switched from their standard and established methods of offering insurance services to new offerings with targeted consumer segmentation. Data-mining systems analyze and store data from databases that contain information on insurance, contracts, and associated data. Data mining can be a combination of machine learning techniques, pattern recognition, statistics, database theory, summarizing and interacting between concepts, and finding interesting patterns automatically from the databases of large organizations whose main mission is to help the decision-making process by extracting knowledge from the data. Data mining has been used to discover hidden knowledge in existing databases in the insurance industry and improve this field [24]. By identifying key variables and their interactions, data mining may assist insurance businesses in making critical decisions and translating the findings into useful and applicable outcomes such as service development, trover analysis, and resource distribution [25].

It should be emphasized that the initial data obtained from the insurance organization were comprehensive and contained no missing data prior to using the k-means approach to cluster the data.

Regarding this matter, obtaining primary data revealed there were 21 776 350 outpatient prescription claims isolated from one another every four years (2016–2019) based on the specific codes and 11 features of the outpatient prescription. The researcher then converted the outpatient prescriptions for each insured using these identification codes. Python software was used to perform basic mathematical operations, such as addition and multiplication, after applying the necessary codes and reviewing the pertinent data to become familiar with the data, identify data quality issues, discover a fundamental view of the data, and identify the subsets required to create hypotheses.

Subsequently, all operations that go into building the final dataset are included in the data preparation step (the data imported from the initial raw data to the modeling tools). No particular order is apparent in the data preparation tasks, which are most likely to be conducted multiple times. As part of this work, features must be registered and chosen, and the data must be transformed and cleaned for use as modeling tools. Prior to processing the extracted information from the data repository, data cleaning, which is a component of data preprocessing before data mining, is crucial. Data cleaning also refers to the process of enhancing data quality by eliminating errors and inconsistencies. The primary objective of data cleaning is to speed up and simplify the extraction process while improving the quality of data in the database [26].

To establish the low-, middle-, and high-risk thresholds, we calculated the interquartile range (IQR) of the cluster sizes. We classify the insureds into three classes—low, middle, and high— to examine particular sets of clusters and categorize the risk assessment of the insured based on the number of prescriptions and medications each insured has. Based on the previously indicated theoretical framework, the risks posed by the insured can be divided into the following categories. Then, to identify the low-, middle-, and high-risk thresholds, we calculated the interquartile range (IQR), which is the area between the third and first quartiles of cluster sizes. We collected insureds with an IQR of 48 388–96 776. We defined low-risk clusters as being below IQR, middle-risk clusters as being within it, and high-risk clusters as being above it.

Data mining techniques are divided into "supervised" and "unsupervised" approaches, according to the most widely used and recognized classification system among professionals. Supervised techniques aim to identify the relationship between an output (dependent) variable and an input variable (features or attributes) (or desired attributes). Unsupervised approaches are utilized when prior knowledge of the dependent variable is not available. Because the dependent variable (optimal co-insurance) in the current study does not yet have known characteristics, and the pertinent data are not yet labeled, the unsupervised method (clustering) was used. Additionally, optimality theories and regression functions related to these monitoring methods were used to estimate optimal co-insurance.

Clustering is one of the most helpful methods for finding groups, determining interest distributions, and identifying patterns in data. The grouping of transactions, observations, or statuses into comparable classes is a data-mining operation known as clustering. A cluster is also a collection of records that are similar to and distinct from those outside the cluster. Clustering does not have a target variable and does not classify, estimate, or predict the value of the target variable [27]. Depending on the type of data, cluster shape, data interval, etc., various clustering approaches are available in the clustering field. These methods, such as fuzzy, hierarchical, and partitional clustering, have diverse working principles and are predicated on specific premises. K-means, defined as follows, is a partitional clustering technique. The clustering will be more accurate if more features are picked because selecting acceptable features is the clustering system's most important decision. Clustering was done in this study using 11 features from the k-means method. The K-means approach is one of the most popular clustering methods. The key factors influencing its appeal are its convenience, efficacy, ease of execution, and practical effectiveness [28].

As a popular technique algorithm, K-means is employed. It uses the distance as the unit of measurement, identifies the K data clusters, computes the average distance, and then returns the starting centroid. The centroid of each cluster provides a description [29].

The objective is to reduce the overall average value by forming disjoint sets of n data points (× 1, × 2,…, xn) into k n sets (S1, S2,…, Sk) (including the square distance from the point to the centroid). Consequently, the optimization objective is to discover:

$${argmin}_s\frac1n\sum_{i=1}^k\sum\nolimits_{x\epsilon s_i}{\Arrowvert x-\mu_i\Arrowvert}^2$$

That ${\mu }_{i}$ represents the average of the points in ${s}_{i}$.

Each point should be assigned to the same cluster as the center closest to it. This is step 1. Choose one initial set at random to serve as the initial centroid.

Step 2. Allocate every point to the cluster that has the same centroid that is nearest to it, indicating that the following formula must be met:

$$s_i^{(t)}=\left\{X:\left\|X-\mu_i^{(t)}\right\|^2\leq\left\|X-\mu_j^{\left(t\right)}\right\|^2\forall_j.1\leq j\leq k\right\}$$

${S}_{i}\cap {S}_{j}=\varphi\;{\forall }_{i}.j\le k$. In other words, a point can only be assigned to one of several centroids if they are all equally far from it.

Step 3: To update the category’s cluster center, the mean value of all items in that category is used.

$${\mu }_{i}^{(t+1)}=\frac{1}{\left|{S}_{i}^{t}\right|}\sum_{{X}_{j}\epsilon {S}_{i}}{X}_{j}$$

Step 4: Assess any changes in the cluster center and objective function values ${\mu }_{i}^{(t+1)}={\mu }^{t}$ ∀i ≤ k. This indicates that the cluster allocation will not change on update if the allowed number of iterations is achieved. If not, proceed to step 2 [30].

In addition to measuring how effectively an observation is clustered, the silhouette coefficient calculates the typical separation between clusters. The mean score for each point included in the dataset provides the foundation for [31]. The proximity of each point in a cluster to those in its neighboring clusters is determined by this index. Therefore, according to this definition:

$${S}_{i}=\frac{{b}_{i}-{a}_{i}}{{\text{max}}\,({b}_{i}.{a}_{i})}$$

where bi is the average distance between the data and the closest cluster inside its own designated cluster, and ai is the average distance between one data point and all other data in the same cluster. Si has a range of -1 to + 1, and a high positive value indicates a strong cluster of data. When Si is close to zero, object i may be considered to be in both clusters.

Next, normalization is performed using min– max scaling, a type of feature scaling. Furthermore, we employ the grid search approach to create the ideal parameters from each cluster’s default parameters, allowing us to compare cluster outcomes based on the best parameter and illuminating the effects of hyper-parameters for future research and better decision-making [32].

As the household income of the insureds could not be calculated, unlike other variables in the health insurance data, the insureds were identified and categorized on the basis of their responses to the codes related to the payment of insurance premiums to calculate the household income using the cost– income questionnaire of the Iranian statistical center. The insureds under other insurances were removed from the samples after identifying the pertinent codes. After all, the steps had been taken, a sample of 38 319 individuals was analyzed to use an ANN technique to estimate the income of the insureds by the Iran Health Insurance Organization.

Artificial neural networks are used for various tasks, including data fitting and attempting to find the optimum fit by modifying the network’s parameters. Generally speaking, neural networks are made up of layers of neurons, each of which connects to the outside world through its inputs and creates the outside world by its outputs [33]. The first step of the neural network is to locate and examine the variables that affect income. The output index is important because the model’s ultimate goal is to estimate the insured’s income using already-existing indicators such as age and sex as well as details about the income of 38 320 insureds that was obtained from the household income-cost questionnaire. Consequently, 19 3552 are generated as estimated annual income. Following these steps, the neural network was trained using various permutations according to the technique used to determine the number of layers and neurons. All of these Python software implementations of the network have the same transfer functions for the hidden and output layers, which are linear and hyperbolic tangent functions, respectively.

Each layer underwent Relu activation, with the first layer including 50 neurons, the second layer containing 50 neurons, the third layer containing batch normalization, the fourth layer containing 50 neurons, the fifth layer containing 20 neurons, and the buried layer containing one neuron. The shap’s neural network’s sensitivity analysis method revealed that 45% of gender and 55% of age were important variables.

Additionally, the MSE for the training data was 8.9 *10 ^12^ and for the test data was 2.9* 10 ^16^ while the learning rate was 10 ^−4^. The high values of these amounts highlight the limitations of the indicators used for an accurate income estimate. This implies that income cannot be accurately anticipated based solely on age and sex.

Finding out how much each insured in each cluster should pay the optimal co-insurance rate is in accordance with economic theories because data mining does not by default optimize the amount of co-insurance of outpatient drug expenses of health insurance insured and only provides the necessary platforms to achieve this goal. Optimization is at the core of economics, as described in the literature on economic theories. The representative household maximizes the benefits of the consumption portfolio within its planning horizon and reduces the expenses of the business enterprise’s objective function within the optimizations. Unconstrained optimizations are essentially irrelevant to economics because they are not defined in the context of employing constrained resources [34].

When a decision-maker has an objective function that he wants to maximize or minimize, the scenario is referred to as constrained optimization. He has n decision variables $\left({x}_{1}{.x}_{2}.\dots .{x}_{n}\right)$ to accomplish this goal, but he is constrained and cannot freely choose the values of all the variables. Since society’s members are not totally free to choose their own insurance coverage against the costs of illness because they are not permitted to have less than a minimum level of support, this insurance should be created in a way that allows a person with rational consumption to choose it. and stop free riding brought on by other people’s actions. From the standpoint of the insured, this issue influences the design of health insurance contracts [35]. When an insured has comprehensive coverage and access to health products with no effective cost, this increases his/her desire to consume to the point where his/her marginal utility is zero (saturation value). The extent to which this comprehensive coverage is not provided depends on the type of medical services offered. There are limits on the amount of consumption, the price of services, and the total expenses (multiplied by the price of consumption) in relation to this amount of compensation (reimbursement), supplied, the providers, and the amount of compensation paid by the insurer [36].

The most general condition, which is typically separated into cost sharing in various ways by the insured, is shown by spending limits. In proportional cost sharing, insurers typically cover the ratio b of all treatment expenses, whereas individuals cover the ratio 1-b = c, or the co-insurance rate. Therefore, the insured’s effective cost of treatment is equal to c of the service provider’s charge, and the insured is concerned when the co-insurance rate rises in relation to the price of effective care [36].

Plans for health insurance are subject to restrictions such as co-insurance worldwide. If it is evident that the lack of co-insurance in this type of insurance leads to excessive consumer moral hazard, health care demand consumers may face significant risk if they choose not to use co-insurance. In actuality, having insurance with co-insurance is preferable to going without insurance or having insurance with full coverage in terms of wellbeing. This problem is congruent with the literature in this area, which demonstrates that optimal (desirable) insurance strikes a compromise between the welfare gained from risk sharing and the lack of welfare caused by moral hazard [35].

The research by Westerhout and Folmer, which was used to estimate the optimal co-insurance for outpatients and inpatients, is the foundation of the model under consideration [35]. There is only one medical product (outpatient pharmaceutical services) in the current study model, which is a best-informed product, and the customers are in various states depending on the kind of risk they are exposed to. Positive out-of-pocket spending; however, prices are lower than the highest co-insurance. This model offers a straightforward framework for the potential continuity of medicinal items and health conditions (depending on risk) (outpatient pharmaceutical services).

The highest utility that can be attained by paying attention to its properties (strictly concave) is related to a risk-averse person if we assume that the individual’s utility depends on the consumption of health care goods (medicines) and is directly dependent on his/her health status (type of risk).

where Z is the amount of health care consumed, C is the number of non-medical services consumed, and Y is the amount of income the consumer earns. Consequently, the utility function is as follows [37]:

1 $$U=C-1/2BC^2+\gamma Z-1/2\delta Z^2\qquad\quad0\leq B<\frac1y.\delta>0$$

According to this study’s practical definition of the variables, maximizing consumer utility aims to either improve the insured’s quality of life or lower drug costs in the household consumption portfolio (implicitly lowering the insured's out of pocket for drug expenses). Consumption of medical services, use of prescription medications, receipt of patient prescriptions, and other care registered for each patient under health insurance, as well as income, including the householder’s income for the householder and other family members.

The range of parameter B ensures that non-medical consumption has a marginal utility, and 1-By is always positive. In addition, it displays patient heterogeneity in terms of risk status and illustrates the differences between various risk levels. In other words, utility is impacted by risk status because it raises the impact of medical care (or, more specifically, the use of drugs).

Equation (1) demonstrates that relative to the marginal utility of obtaining medical treatment, according to Latmer and Finkelstein’s studies on the separability of preferences for medical and non-medical service consumption, the marginal utility of non-medical service consumption declines as the health state worsens [35]. These findings change the consumption portfolio toward non-medical products and, in the event of health worsening, toward healthcare, which is similar to the design of our model in this study (drug consumption).

The value of parameter b, which is $0\le b\le 1$, defines the co-insurance rate (current pharmaceutical co-insurance premium for each prescription). where represents the cost of the healthcare service provider (drug costs), bt represents the out-of-pocket cost of drugs, and p represents the monthly insurance premium that each family member must pay.

2 $$\begin{array}{cc}\begin{array}{cc}{\text{c}}={\text{y}}-{\text{p}}-{\text{btz}}&\qquad 0\le {\text{z}}\le \frac{{\text{m}}}{{\text{bt}}}\\ {\text{c}}={\text{y}}-{\text{p}}-{\text{m}}& \frac{{\text{m}}}{{\text{bt}}}\le {\text{z}}\end{array}& \end{array}$$

The consumer’s budget is limited in a non-linear manner based on the maximum co-insurance contribution, or m. According to Eq. (2), where the value of the parameter that indicates the consumer’s health status is present, the utility function (assumption of optimality) is maximized by the consumer (insured risk).The parameter $\upgamma$ can only receive three values as a result of this.

First, if ${z}_{1}=0$, then $\upgamma$ can be ${\gamma }_{1}$. This is equivalent to the healthy consumer who has no need for medical care.

Second, if ${{\gamma }_{2}>\gamma }_{1}$, ${\gamma }_{2}=bt\left(1-B\left(y-p\right)\right)>0$, then $\upgamma$ can be ${\gamma }_{2}$. The patient who requires medical attention is also a part of this case, and his co-insurance payments are fully positive but less than the maximum m. Greater expenses translate into higher co-insurance payments for this patient.

Third, $\upgamma$ can be (possible) in the scenario of ${{\gamma }_{3}>\gamma }_{2}$, which denotes that there is a significant demand for medical services so that the customer can make the largest co-insurance payments. The following are the exogenous probability ($\pi$) and health care demand linked to these three states:

3 $$\begin{array}{llll}{\pi }_{1}\ge 0.{\pi }_{2}>0.{\pi }_{3}\ge 0\\{Z}_{1}=0\\{Z}_{2}=\frac{{\gamma }_{2}-bt(1-B\left(y-p\right))}{\delta +B{(bt)}^{2}}\\{Z}_{3}=\frac{{\gamma }_{3}}{\delta }\end{array}$$

In addition, it is assumed that the health insurance market is totally competitive and has no administrative expenses, an assumption that is entirely comparable to universal (national) insurance. Because we assume that the insured’s circumstances are the same before the occurrence of health shocks, adverse selection is not a factor in our study or equations. As a result, the health insurance premium is determined by subtracting the co-insurance payments from the medical costs:

4 $$p={\pi }_{2}-\left(1-b\right)t{z}_{2}+{\pi }_{3}\left(t{z}_{3}-m\right)$$

The necessary condition for the optimal point by differentiating from E(u), which maximizes the optimal co-insurance rate of Eq. (1), is finally expressed using the first- and second-order conditions of the expected utility function.

Selecting the value of $\varphi$ that maximizes insured's expected benefit becomes selecting the optimal co-insurance amount for health insurance:

5 $${b}^{*}=\frac{\varphi \varepsilon }{\left(1-\varphi \right)+\varphi \varepsilon }$$

which in $\varphi$ and ${\varepsilon }_{p}$ are equal to:

$$\begin{array}{c}\varphi=\frac{\pi_1-(\beta\left(y-p\right))}{1-\beta(y-p-btz_2)}+\pi_2+\frac{\pi_3(1-\beta\left(y-p-m\right))}{1-\beta(y-p-btz_2)}\\\varepsilon_p=\frac{-2\gamma_2\beta\left(bt\right)^2-\left(1-\beta\left(y-p\right)\right)bt(\delta-\beta\left(bt\right)^2)}{(\gamma_2-\left(1-\beta\left(y-p\right)\right)bt)(\delta+\beta\left(bt\right)^2)}\end{array}$$

Solving the aforementioned equations and corresponding estimates—which are derived by converting these formulas in Python software to corresponding codes—results in the optimal co-insurance value.

Given that each study needs some presuppositions to analyze and maximize the intended value, we have no role for suppliers (health insurance firms) or full information on this segment of consumers regarding their health state after experiencing health shock. Instead, we suggest a different theory that bases its calculations on the estimation of optimal co-insurance and the assessment of appropriate models and examines the cost shift of the health insurance organization after estimating the optimal rate of pharmaceutical co-insurance. This significance was determined by simply subtracting the entire drug cost covered by insurance before and following the use of the best co-insurance.

Toward the end, due to the fact that the data are based on the Iranian Rial, after all clustering and income estimation procedures have been completed, the cost and income variables have all been adjusted in accordance with the central bank’s stated average exchange rate for the four years 2016–2019 [9]. (1 USD = 12,448.14 Rial).

### Patient and public involvement

This study was not conducted with patients or the public involved in the design, execution, reporting, or dissemination strategies.

## Results

After cleansing the data, there were 21 776 350 outpatient prescription claims. Then, for clustering using the k-means method based on 11 features, these claims were converted into 193,553 individuals over the course of 4 years. Afterwards, we established low, middle, and high-risk classes based on the IQR of 48 388–96 776 insureds. These insureds were split into three clusters according to the silhouette coefficient for each class. The economic optimal co-insurance formula developed by Folmer and Westerhut utility model was used to calculate the optimal co-insurance rate for all insureds in each cluster and class.

Due to the fact that co-insurance is a portion of the treatment cost that the insured must pay at the time of receiving services, Table 1 displays the estimated optimal co-insurance for each cluster of insureds in the three classes of low, middle, and high risk. Inferring from these findings, the optimal co-insurance rate is 0.81 for the low-risk class in the first cluster, which has 21,779 insureds. In addition, for the second and third clusters of the low-risk class, this value is 0.76 and 0.84, respectively.

**Table 1:** Optimal co-insurance rate for each cluster's insureds

**Class**	**Cluster**	**Number of insured**	**Optimal-coinsurance**
**Low risk**	Cluster 1	21,779	0.81
	Cluster 2	7170	0.76
	Cluster 3	19,419	0.84
**Middle risk**	Cluster 1	48,348	1
	Cluster 2	23,321	1
	Cluster 3	25,107	1
**High risk**	Cluster 1	14,037	0.38
	Cluster 2	28,504	0.45
	Cluster 3	5847	0.42

Contrary to the insureds in the low-risk and high-risk classes, all of the insureds in the three clusters of the middle risk class are required to cover all outpatient prescription costs in accordance with the calculated 1 co-insurance optimal for them.

As well the insureds should pay 38, 45 and 42 percent of the outpatient prescription expenses, respectively, for the first, second, and third clusters of the high-risk class, which is also identical to the interpretation of the optimal co-insurance for the first cluster of the low-risk class. Cost variations for insurers (US$).

Table 2 is based on the supposition that if the insureds of each cluster and class are subjected to the optimal co-insurance, how much will the health insurance organization’s (insurer's) costs change? By adopting the optimal co-insurance, for instance, in the first through third cluster from the low-risk class, the health insurance organization will make profits of $ 3,130,463, $3,451,194, and $1,069,859 as a result. Middle risk class’s profits are $29,239,815, $13,863,810, and $14,573,432; high risk class generates revenue of $4,722,099, $6,339,317, and $19,627,062.

**Table 2:** Optimal co-insurance rate effect on changes in insurer cost based on dollars

**Class**	**Cluster**	**Number of insured**	**Cost variations for insurers(US$)**
**Low risk**	Cluster 1	21,799	3,130,463
	Cluster 2	7170	3,451,194
	Cluster 3	19,419	1,069,859
**Middle risk**	Cluster 1	48,348	29,239,815
	Cluster 2	23,321	13,863,810
	Cluster 3	25,107	14,573,432
**High risk**	Cluster 1	14,037	4,722,099
	Cluster 2	28,504	6,339,317
	Cluster 3	5847	19,627,062

## Discussion

The present study transformed 21 776 350 outpatient prescription claims from health insurance organizations into 193 552 individuals. based on the IQR of 48 388–96 776 insureds, they were divided into low, middle, and high-risk classes. Then, using the k-means approach, silhouette coefficients with 11 features were split into three clusters. The four-year period’s first through third clusters of data revealed 21,799, 7170, and 19,419 insureds in the low-risk class. While 48,348, 23,321, and 25,107 covered insureds were in the middle risk class. 14,037,28,504, and 58,747 insureds belonged to the high risk class.

With regard to risk, Wang et al. employed a value-based strategy to treat heart disease in patients with low- and high-risk health. They found that compared with preventing heart attacks in high-risk patients, preventing the conversion of low-risk patients into high-risk patients is more crucial in lowering total expenditures [10].

After laying the groundwork for the clustering method, optimization was achieved using the Westerhut and Folmer utility model, and the optimal co-insurance for each cluster was estimated in accordance with the insured features in each cluster. For the first through third cluster in the low-risk class, the co-insurance rate ranges from 0.81, 0.76, and 0.84, whereas for the same clusters in the high-risk class, it varies from 0.38, 0.45, 0.42. This rate is one for all clusters falling into the middle risk class. That is, insureds with similar and identical features, such as the total average number of medicines, the total average number of medicines for acute and chronic disease, the total average insurance paid, the total average franchise (co-insurance cost paid by insured), the total average number of prescriptions, the total average insurance paid and deductions, the total average income (estimated income for each insured using artificial neural network), and the total average deduction (deductions per prescription), must pay 81%,%76 and %84 of their costs when receiving a drug prescription.( To see the details of clustering and clusters, see the paper K-means clustering of outpatient prescription claims for health insured in Iran) [38].

The study by Mahlich et al. compared the medical developments in Japanese patients with rheumatoid arthritis who were 70 years old before and after 2014, when the co-insurance rate was reduced from 30 to 20%. They found that out of 7343 patients with rheumatoid arthritis, 67% (4905) had a 20% reduction in co-insurance [21].

Co-insurance rates that enhance the welfare of the diverse patient population at risk of cardiovascular disease are sought for by Gregory et al. They discovered that lowering co-insurance rates can help individuals who are at high risk for cardiovascular disease comply with treatment plans more consistently and have better health outcomes [39]. In a circumstance where everyone has an equal risk profile, Pauli et al. illustrate that the co-insurance rate should differ and be greater for medical services that have varied levels of price responsiveness or price elasticity of demand. According to a different approach termed "value-based cost-sharing," co-insurance needs to be reduced for therapies that have better cost–benefit ratios [37].

In order to promote medication adherence and Health Outcomes, Wang et al. employed a value-based strategy for treating heart disease in which members with low- and high-risk health insurance had distinct parameters for cost-sharing. In this study, the Markov chain approach is utilized to model changes in health conditions in the context of designing health insurance, and sensitivity analysis is used to examine how important parameters affect optimal co-insurance levels. The final finding of this study demonstrated that, in comparison to preventing heart attacks in high-risk patients, preventing the conversion of low-risk patients into high-risk patients is more crucial in lowering total expenditures. Moreover, when heart attack reduction rates are less than 14%, cost reductions based on a value-based approach and optimal cost-sharing levels are particularly sensitive to drug effectiveness [40].

Joyce used a retrospective analysis to investigate the effects of benefit package changes, such as multi-tiered formularies and required generic replacement, on the total cost to insurance providers for generic and brand medications and direct out-of-pocket payments to beneficiaries. Data on 420,786 employees of major corporations (those with 25 or more employees) between the ages of 18 and 64 who had access to outpatient drug health insurance benefits were gathered between 1997 and 1999. The average annual drug expense per person for a tier 1 plan with a $5 co-payment for all prescription medications is $725. The average annual prescription expenditure would be reduced from $725 to $563 per member by doubling co-payments to $10 for all medications. The cost of the tier 2 plan would drop from $678 to $455 by doubling the co-payment from $5 for generic pharmaceuticals and $10 for brand drugs to $10 for generic drugs and $20 for brand drugs. A tier 2 plan with a $30 copayment for non-preferred brand medications reduces overall prescription costs by 4%. The requirement of generics in tier 2 plans has resulted in an 8% decrease in prescription expenditures, while the gap in recipients' out-of-pocket expenses has grown from 17.6% to 25.6 percent. The final step is to lower all program payment costs as well as the total cost of drugs for the employer’s insurance coverage by introducing an extra level of co-payments or raising the co-insurance amount. Health insurance plans have considerably profited from the decline in prescription prices because it has raised the proportion of direct payment costs that patients bear out of their own pockets [41].

How much profit can the insurance organization make with regard to this subject if the best co-insurance strategy is used, For the low-risk class, the insurance company makes profits of $3,130,463 and $3,451,194 from the first through second clusters, but only $1,069,859 from the third cluster for the same class. According to the results table, the insured in all three clusters of the middle class of risks pay the full cost of outpatient prescriptions based on the optimal co-insurance, which results in the largest profit for the health insurer. Regarding the first cluster's insured, it is $29,239,815. With a value of $19,627,062, the third cluster of the high-risk class has the second-highest profit amount. The first and second clusters in the same class had substantially lower profits of $4,722,099 and $6,339,317, respectively.

An investigation into the financial effects of the two-tier payment system on the annual cost of pharmaceutical benefits in the National Health Insurance of Korea revealed that if there is no change in the use of original and generic medications, the total yearly drug cost declines by 1.3%; if there is a change in the use of original and generic medications, the cost decreases to 4.3% [42].

As a result of the current investigation’s findings, it is possible to draw the conclusion that resources and costs can be moved using a variety of strategies, including variable co-insurance. When the recipient of a service cannot cover the high cost of that service because they cannot afford it, resources and expenses are incurred [43]. Therefore, insureds in the middle risk class release the most funds for the health insurance company, which may then be distributed to other patients/insureds in need of healthcare. Thus, vertical equity in the health sector will be promoted.

One advantage of this study over previous studies is that it attempted to account for all the factors influencing the optimal co-insurance rate, such as the insured’s demographics, factors relating to costs and the number of prescriptions, and changes in the insurer’s expenses, which were absent from previous studies. Furthermore, by employing the k-means approach for the optimal clustering of the insureds, additional individuals with the same features as the clustered individuals can be assigned to one of the three classes in order to calculate their optimal co-insurance, with the results being simple to apply to policymakers.

### Suggestions for future research

Many assumptions are necessary for any comprehensive investigation; however, this study regrettably did not pay attention to them. According to economic theories, it is necessary to take into account both the supply and demand sides to arrive at an equilibrium price or rate. Based on the demander’s best estimate, only changes in the insurer’s costs were obtained in this study; supplier-affecting variables were excluded. Therefore, it can be proposed that in some research, the impact of the supplier (insurer) should be considered more thoroughly. Additionally, in order to establish access and fairness in health care services for individuals based on the objectives of health systems, the optimal co-insurance rate for testing, diagnostic, and inpatient services should be estimated independently.

## Conclusion

Without adding financial burden and developing a brilliant system for calculating co-insurance, equity cannot be improved. The access of vulnerable groups to drugs with catastrophic costs grows as resources are redistributed and moved from cheap and widely used drugs to pricey and seldom used medications. This study aims to demonstrate how the health insurance organization can change co-insurance from a uniform state and, as a result of changes in drug costs and expenses as well as insured characteristics, use health insurance resources in a way that is appropriate for people whose co-insurance is high (people who are likely to experience catastrophic expenses) and those who do not consider the economic consequences of their behavior (moral hazard). To meet the two aforementioned aims while maintaining access levels and patient health costs, it is important to obtain optimal co-insurance.

## Data Availability

Data are available upon reasonable request. shekoufehmomahhed@gmail.com will be responsible for any information about the data.
